# Primary care physicians’ experiences of video and online chat consultations: a qualitative descriptive study

**DOI:** 10.1080/02813432.2024.2391406

**Published:** 2024-08-18

**Authors:** Kaisa Kujansivu, Elina Tolvanen, Mervi Kautto, Tuomas H. Koskela

**Affiliations:** a Department of General Practice, Tampere University, Tampere, Finland; b Pihlajalinna, Tampere, Finland; c The Wellbeing Services County of Pirkanmaa, Tampere, Finland

**Keywords:** remote consultation, video consultation, chat consultation, telehealth, telemedicine, primary health care, physician

## Abstract

**Objectives:**

To explore the perceptions and views of remote consultations and patient care of primary care physicians (PCPs) who work remotely regularly and have experience performing remote consultations.

**Design:**

A qualitative study using thematic analysis.

**Setting:**

Four online focus group interviews with 17 PCPs, working with one private health care provider, with public and private primary care patients.

**Subjects:**

PCPs who had performed video or online chat consultations with primary care patients for at least 6 months.

**Main outcome measurements:**

PCPs’ perceptions and views working remotely in online chat and video consultations.

**Results:**

Two main themes describing how PCPs perceived remote consultations emerged: 1) remote consultations have an impact on the way physicians work, and 2) remote consultations have an impact on the service system and patients. The subthemes of the first main theme included the physicians’ new way of working, impacts on physicians’ well-being, and impacts on communication and physician competences. The subthemes for the second main theme were the importance of smoothness of services for the patients, patient suitability, and technical liabilities.

**Conclusion:**

Remote consultations provide PCPs with a new way of working that could improve work-life balance. However, it is important to maintain sufficient clinical competence through versatile work. Digital consultations can make contacting healthcare smoother and easier for patients if the patient selection is performed carefully. Online chat seems suitable for singular contacts and simple issues, but remote consultations could be used to sustain continuity of care.

## KEY POINTS

- PCPs perceived that remote consultations had an impact on the PCP’s work and the service system
- PCPs felt that remote consultations gave them a new way of working and had a positive impact on their work
- PCPs perceived that working remotely required skills from the PCP, such as sufficient previous clinical experience in face-to-face consultations and communication skills
- PCPs felt that patient selection for remote consultations was important
- PCPs considered that lack of continuity of care limited the issues that could be dealt with in a remote consultation.
- PCPs felt that technical liabilities must be considered.

## Introduction

The work of a primary care physician (PCP) is undergoing change as different digital services have increased in health care in recent years [1]. In Finland, remote primary care services are more common compared to the EU average [2]. In Finland, as an alternative to telephone consultations, video, and online chat consultations have been increasingly used in primary care services in remote consultations [1]. Performing consultations remotely instead of traditionally face-to-face (F2F) provides PCPs new opportunities for physician work in primary care. PCPs have perceived remote consultations to be convenient and safe, especially during the COVID-19 pandemic [3,4]. In addition, PCPs have noted remote consultations hold the potential to reduce the workload of physicians and offer flexibility and high autonomy [5,6], although there have also been opposing findings with increased workload [7]. PCPs have also been satisfied with the smoothness and ease of use for the patients as well as the reduced time in contact with health care [4, 8,9].

In addition, performing video consultations has an impact on the assessment of the patient’s condition and the communication between the patient and PCP [5]. Considering communication, PCPs have stated that important non-verbal communication is lost in remote consultations [4]. This loss of non-verbal communication can also affect clinical decision-making [4, 10,11]. In addition, communicating by writing is challenging [12]. Moreover, lack of physical examination and loss of physical presence and touch could affect the physician-patient relationship [13]. Video consultations have been considered most suitable for situations where continuity of care is present [14].

Physicians have noted that remote consultations are not suitable for every complaint and are most suitable for complaints where physical examination is not significant, such as issues concerning mental issues, mild infections, and follow-up visits [5, 10, 14,15]. In addition, remote consultations are not accessible to all patient groups [4, 13, 16,17]. Comparing different types of remote consultation, a telephone consultation has been considered sufficient for many problems [18]. However, by improving communication, video consultations have seemed superior to telephone consultations for mobility and mental health problems [19]. Video consultations have the benefit of saving time [13, 20] and reducing the number of visits [21], but there are also controversial perceptions that video consultations are not saving time and are less effective as F2F consultations [22].

Remote consultations bring up the need for additional skills as the physician has fewer diagnostic clues. Previous studies have shown physicians calling for training before attending remote consultations [3] but more importantly for strong clinical experience from F2F consultations [5, 22].

Technical liabilities are to be considered regarding the service system and the physicians’ work [4, 10]. Physicians have felt that remote consultations need to be more reliable and seamlessly integrated into the service system [19]. The implementation of different digital solutions should not increase PCPs’ workload [7, 13]. In addition, easy access to care can be problematic [5] and could lead to varying quality and rising costs [23].

Even though remote consultations seem to have potential benefits, their status is still unestablished. Remote consultations seem to have a lack of dignity among physicians, and they have been suggested “not to be the real work of a physician” [5,6, 24].

This study aimed to explore the perceptions and views of remote consultations and patient care of primary care physicians (PCPs) who work remotely regularly and have experience performing remote consultations.

We focused especially on experiences with video and online chat consultations and the benefits and challenges the PCPs have faced.

## Methods

The methodological orientation of this study is based on the phenomenological theory of the description of the participants’ personal subjective experiences. With a phenomenological view [25], we wanted to study PCPS individual perspectives on experiences of remote consultations. We used thematic analysis, as it enabled us to identify patterns of meaning across a set of data and explore PCPs’ experiences and reflections on the clinical work they presented.

The research team: Kaisa Kujansivu (KK, GP) and Mervi Kautto (MK, GP) conducted the focus group interviews. In addition, Tuomas Koskela (TK, GP, Associate professor of General Practice) and Elina Tolvanen (ET, GP, PhD) were involved with designing the study and interpreting the data. KK worked as a chief medical officer at Pihlajalinna eHealth services before, during, and after the interviews. MK worked as a clinical lecturer at Tampere University during the interviews and partly in clinical work for Pihlajalinna. MK performed the interviews and KK was present as an observer. KK had prior contact with all the PCPs before the interviews by working in the same unit. KK also held a current or prior supervisory position over some of the PCPs participating.

Health care services in Finland are fragmented, with wellbeing services counties, the health insurance system and employers involved [26]. Pihlajalinna Plc is one of the three largest private healthcare service providers in Finland, providing primary health care services to the private and public sectors. The PCPs in this study were recruited from the Pihlajalinna eHealth unit. PCPs with strong previous experience in remote appointments *via* chat or video were invited to focus groups by group and private messages (e-mail, Microsoft Teams, and WhatsApp). The characteristics of the PCPs are shown in Table 1. The inclusion criteria were either chat or video consultation experience for at least six months prior to the interviews. Twenty PCPs were scheduled for four different focus groups, and altogether three PCPs cancelled just before the interview. The patients they had treated remotely were either primary care patients from a certain public health care centre and/or primary care patients from occupational health care in a nationwide service.

**Table 1.** Characteristics of the participants.

	Classification		n	%
Education	Licentiate of medicine		12	70.6
	Specialist in general practice		2	11.7
	Specialist in occupational health care		2	11.7
	Other specialist		1	5.8
Clinical experience	<3 years		1	5.8
	3–9 years		10	58.8
	≥10 years		6	35.3
Experience in remote consultations	6–12 months		1	5.8
	12–24 months		6	35.3
	over 24 months		10	58.8
Remote hours/week	4 h		2	11.7
	8 h		6	35.3
	16 h		4	23.5
	24 h		2	11.7
	32 h		2	11.7
	40 h		1	5.8
Main type of remote consultation	Chat		9	52.9
	Video		8	47.1
Main form of clinical work	F2F consultations		10	58.8
	Remote consultations		7	41.2

The discussion in the groups was liberated concerning different forms of remote consultations in general. In addition to online chat and video consultations, all PCPs also performed telephone consultations. In this article we use the term remote consultation in general, and online chat and video consultations are mentioned separately when it is considered necessary.

Finally, we had 17 PCPs divided into four focus groups (one group with five participants and three groups with four participants). PCPs with experience in online chat and video consultations were involved in all the groups. The interviews were performed in Microsoft Teams meetings since the physicians were from different regions. It has also been suggested that face-to-face interviews are only marginally superior to video interviews [37]. The interviews were arranged from the 17^th^ of May to the 7^th^ of June in 2022. The first interview was also a pilot for the question frame, and no changes were made after the first interview. Besides the participating PCPs and two researchers, no one else was present in the interviews. A semi-structured interview frame was used in the interviews. The discussion was unrestricted, and the interviewer ensured that all the topics were covered according to the question frame (Appendix 1). The interviews were recorded with Microsoft Teams and the recordings were transcribed. The interviews lasted from 53 to 78 min, and 37 pages of transcription were produced. The transcriptions were anonymized and analysed by all four research team members. The material was analysed and coded by all four research group members first separately, and the main researcher merged the codes.

The data were coded according to the thematic analysis process [27]. We organized the data into sub-themes and themes first individually and then the main researcher emerged the codes. Each hierarchical level was discussed in F2F meetings. We combined the codes into overarching themes that aimed to depict the data according to the study aims and research questions. Atlas.ti software and Microsoft Excel were used for the coding and grouping of the data [28]. The data were found to be saturated after four interviews and no more interviews were considered to be needed.

Finally, we looked back at the raw data to see how it supported the themes and our overarching theoretical perspective. We looked for original data citations that supported the analytical themes. We checked that every quote and code could be assigned to at least one theme. We combined the advantages and disadvantages of remote consultations on a separate table and suitable and non-suitable issues on another table. COREQ [29] checklist was used as our reporting guidelines.

## Results

The characteristics of the participants are shown in Table 1.

**Figure 1.**
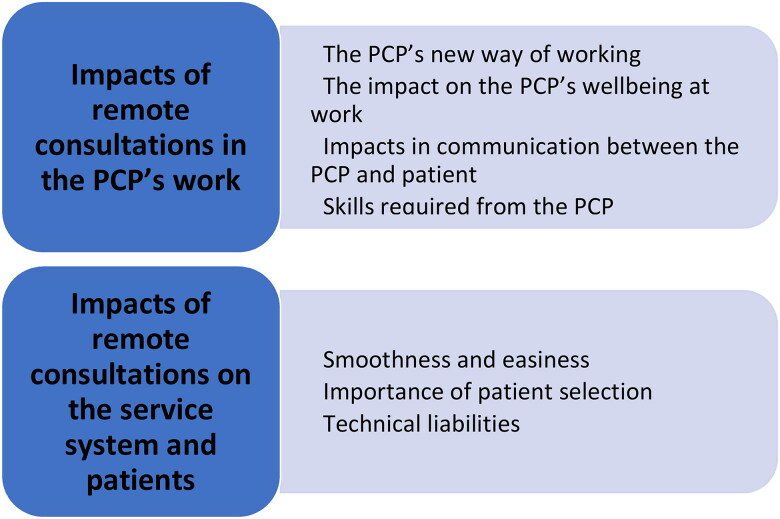
Main themes and subthemes.

Two main themes emerged from the data: 1) the impacts of remote consultations on the practical work of the PCP, and 2) the impact of the remote consultations on the service system and the patients. These two main themes were divided into subthemes (Figure 1).

### The impacts of remote consultations in the practical work of the PCP

In the interviews, several themes emerged concerning the PCP’s practical work.

### The PCP’s new way of working

PCPs perceived that remote consultations brought a new way of working when they were able to work remotely from home. However, some of the PCPs felt that treating patients F2F is the real work of a physician, and working only remotely makes the clinical approach narrow. The PCPs felt that in remote work consultation is easy between professionals. However, the PCPs missed the working community while working remotely.

"But if you did it as your only work, the work would be very narrow. I’ve noticed that meeting people really is, after all, the real work of a physician. I clearly enjoy it more and miss it. I couldn’t continue only in the chat."

Time management can also be an issue in remote work. Especially in online chat, the challenges of time management are related to the challenges of self-management, and how many patients one picks from the queue at the same time.

"In chat, the time management challenges are clearly such self-management challenges /…/ then it’s up to you how many you hoard for yourself at once, with how many patients you jump between those conversations. Then it sometimes backfires when a more difficult case arises; I think that I’m going to spin a fourth patient here /…/, and then it’s something much more demanding, so you can watch yourself carefully so that you don’t get too greedy."

### The impact on the PCP’s wellbeing at work

Most of the participants felt that working remotely is less stressful and burdensome, and it brings more freedom and balance to working days, especially if they have small children. Time is saved when working from home and not commuting. Some participants felt that working remotely had saved them from burnout. The PCPs like to work remotely part-time, but many of them would not want to work remotely full-time. They felt a variation in workdays increases their wellbeing at work.

"Everyone there (at the health centre) seems to be more or less burnt out, under an awful lot of demands, especially those that remain in the public sector. If you work remotely, it relieves the day a great deal, and you feel like you can still do more than you would in ordinary practice. I would definitely say that everyone should work remotely for a day or two a week."

Furthermore, remote work can also be stressful, but the burden differs from F2F consultation days. Many participants felt that facing patients remotely is less stressful than F2F, even if they had more contacts. Working remotely seemed to have fewer distractions. The PCPs also felt content when they were able to help patients quickly with their problems.

"Although it may be that there are even more patient contacts and you feel in a hurry and stressed in another way, the versatility is good."

### Impact on communication between the PCP and patient

The PCPs felt remote consultations have impacts on communication. Different aspects of these effects on communication in video and online chat consultations are shown in Table 2 *(*Table 2*).* Nevertheless, the PCPs felt that communication was typical for the patient regardless of the type of appointment. In online chat conversations, consideration is also required in expression, patient can easilly feel offended in chat, especially if the physician uses punctuation marks.

**Table 2.** PCPs’ statements on communication in remote consultations.

PCPs’ statements on communicating online
**Overall observations on communicating online**
Anxiety becomes more apparent in F2F appointments
Interaction is typical for the patient regardless of the type of appointment
It is safer to face an aggressive patient in a remote appointment
The "door handle questions" are left out of remote consultations
The patient may need a summary from the remote appointment
Young people are more relaxed in remote appointments
**Communication in chat consultations**
Chat interaction is influenced by the patient’s age, background, and personal way of communicating
Chat requires communication skills from the patient
The encounter is more superficial
Excessive directness is harsh in chat
Achieving good communication in chat is possible
Misunderstanding in the interpretation of terms is common
Patients can get angry if their matter isn’t treated remotely
The start and end are vaguer
Using humor is possible
Extensive writing is possible
Accuracy of expression is needed
A neutral start is needed
Communication skills evolve while working in chat consultations
**Communication in video consultations**
Gesture language is limited with pain and psychiatric patients
Patients can be nervous and bring fewer things to video appointments
Patients’ unaccustomedness to the use of video affects communication
Communication is similar to F2F consultations
Communicating on video is not as good as communicating F2F
Communication succeeds better on video than in other remote appointments
Video brings more to communication but not much more to status
Dispelling worries is easier F2F than over video
Working of technology worries patients

Communicating by writing had challenges in the interpretation and understanding of terms, as well as the transmission of empathy:

"So the fact that in chat, the start and end of the reception are somehow much vaguer, when it starts and when it ends, and in some ways, it’s harder to say what kind of interaction we’re in. There are no facial expressions to conclude anything from, there is no tone of voice to deduce anything."

The PCPs felt that video had added value to the communication but not to the physical examination. With video, it was possible to observe more nonverbal communication clues. Some participants felt that *via* video the interaction was as good as in F2F consultations, but some felt it was not as good, e.g. for pain, psychiatric, or somatizing patients.

"Body language is a bit narrower when watching a video compared to a person sitting in the same space if I’m thinking about psychiatric patients, and maybe to some extent pain patients as well."

Sometimes the patients – especially the elderly – seemed to be nervous at the beginning of the video consultations, but in the end, they seemed to be satisfied with the contact after all.

"On the other hand, in the video you can already see in the patient’s face that, and usually the patients also say, oh, this is handy and yes it worked, even though there were some doubts beforehand about whether this would even work with these devices and everything and whether anything would come of it.”

### Skills required for a PCP

A new way of working also requires additional competences from the PCP. In addition, working remotely requires training. Participants noted that it is important to form boundaries and be able to say no to certain complaints to keep the consultations safe. The participants call especially for decision-making skills in remote consultations.

"That’s why I would highlight that when there is a shortage of employees, experience, and competence must be emphasized in recruitment. They should have extensive experience in treating different conditions, which also enables them to assess what can and cannot be treated. They should also have the backbone to tell the patient that this cannot be done. Or that your competence is not sufficient, that you don’t know the issue well enough to make a decision."

Furthermore, the participants called for clinical experience. The PCPs felt that clinical experience defines which diagnoses one can set remotely. A physical examination is not always necessary, and the diagnosis can be based on interviewing the patient and the medical history. They found it critical that PCPs have strong previous experience in F2F consultations to enable them to make decisions remotely. With sufficient clinical experience, the PCPs felt more certain and could speed up remote consultations, as decisions were made with incomplete information without a physical examination.

"You must have seen those situations in the emergency room, especially those that patients have gotten worse. /…/ Clinical experience is needed to assess remotely whether the patient should be referred to the emergency department instantly. I wouldn’t choose a newcomer for this job. I somehow feel, although it’s kind of an easy job, it takes a lot of responsibility to make decisions with incomplete information."

Knowing the service system is a challenge, especially in a nationwide remote service, but orientation and support staff can fill these gaps, at least partially. The participants felt that if the service system knowledge is insufficient, it might add to the use of resources, e.g. the need to guide the patient to a nurse if the PCP does not know enough about local practices.

"Yes, I definitely see that it is important to know the field where you operate, with what resources, how many forks you have in the box, and what fork you use in a certain situation. But you also know how to ask questions and open your mouth."

### The impact of remote consultations on the service system and the patient

#### Smoothness and ease

The interviewed PCPs had received some instant feedback from their patients about online chat and video consultations, and based on this feedback, the patients seemed to be satisfied with the smoothness and ease. Remote consultations save patients time and effort when they do not need to move anywhere, especially during workdays.

"Yes, that easy access is really essential, and for a lot of people, it even comes as a surprise that when you contact them in the chat, in three minutes the prescription for the antibiotics for a UTI is delivered to the pharmacy. They wonder how it can be that easy, and it could happen on a car trip, or a train ride, and you don’t even have to use voice or video. People chat from pretty strange places, but that’s definitely the advantage of chat."

The PCPs also felt that it is easier for some patients to discuss difficult issues online than F2F, and the easy access allows the patient to contact health care more easily in a situation where they normally would not be able to book a F2F appointment.

"But on the other hand, it feels like patients may have the courage to talk about such difficult things when they are in a safe environment in their own homes. It could be more relaxed for them to be there than to be at the practice, so maybe you can get more out of the patients once in a while.”

However, the PCPs also recognized that easy access also adds to demand. They have seen a lot of non-medical advice on chat and have faced issues that would have resolved themselves before the patient would have been able to come to an F2F consultation. In chat consultations, the patients seemed to have gotten used to the fast contacts and were frustrated if they needed to queue online for a few minutes more.

“On the other hand, you will notice over time that those who are used to using chat will be completely frustrated if they have already had to wait for ten minutes. We are teaching them that you get everything fast.”

### Importance of patient selection

The PCPs felt that remote consultations could improve undertreatment regionally but also provide a possibility for overtreatment in some cases. Not being able to do a physical examination increases the risk of over-examining with, e.g. laboratory tests.

"After all, these remote services have enabled more equal services for patients regionally. In localities where the availability of care is worse, especially in face-to-face appointments, patients are able to book remote appointments with a doctor in another location and get help for certain ailments suitable for remote appointments, thereby saving those F2F appointments for others."

The PCPs also felt that online chat communication and the treatment manners of remote consultations should not be generalized to all care. Chat is ideal for more simple issues and singular contacts, but not for everything. The participants considered that treating suitable issues in remote consultations saves F2F appointments for those who need them. In Table 3, we have collated which issues the PCPs noted are usually suitable for remote consultations and which they think are not (Table 3). In the PCPs’ opinion, suitability depends on the remote consultation type and how much time one needs to solve the issue. Online chat consultations have time pressures when taking care of multiple patients simultaneously.

**Table 3.** PCP’s perspective on suitable and unsuitable issues in remote consultations.

Suitable issues for remote consultations	Non-suitable issues for remote consultations
Allergy symptoms	Acute neurological symptoms
Control visits	Complex issues of multimorbid patients, except familiar patients
Evaluation of medication and prescription renewals	Gynecological disorders
Evaluation of sick leave	Injuries
Infection symptoms	Medicines affecting mainly on central nervous system
Insomnia	Multimorbidity
Limited children’s issues	New musculosceletal symptoms
Migraine/Headache	Small children
Musculoskeletal disorders, if recent physical examination available	Somatizing patient
Preliminary consultation before F2F appointment	Stomach aches
Simple matters of healthy patients	Working ability evaluation of a new patient
Stomach flu	
Urinary tract infections (women)	
Whole patient material with sufficient preliminary information	
Working ability evaluation and occupational health negotiation of familiar patients

“Still, there have been issues that I have felt could be handled remotely, but not under this time pressure. It is always somewhat determined by the current queue situation. It has felt like a kind of burden.”

Complex issues demanding physical examination were not perceived as suitable for remote consultations. The most suitable issues for remote consultations were considered, e.g. control visits and the evaluation of medication and prescription renewals. Multimorbidity, acute symptoms, and injuries were considered unsuitable unless the patient was known previously by the PCP. With familiar patients, also complex issues were considered possible to solve remotely.

Furthermore, the type of remote consultation influences suitability. Table 4 shows the issues divided into suitability for chat or video based on interviews (Table 4). Table 4 also lists PCPs’ assessments of other factors affecting suitability. The PCPs felt they had more time to focus on the patient’s problem and could manage more complex issues in video consultations. In online chat, it is possible to solve issues with photos available. In the PCPs’ opinion, a lack of resources elsewhere directs patients to chat about unsuitable issues. They felt that the PCP always has the last word on suitability. The PCPs also felt that the patient rarely knows whether the matter is suitable for remote consultation, and what their matter medically requires.

**Table 4.** PCPs’ assessments of the suitability of different forms of remote consultations.

Special suitability for chat	Special suitability for video	Other factors affecting suitability
Acne (photo available)	Children’s issues	Lack of resources elsewhere directs patients to chat on unsuitable issues
Small bite injuries (photo available)	Evaluation of larger entities	Suitability is affected by the form of remote consultation (chat/video)
Eye infections (conjuctivitis)	Mental health visits	The physician decides on the suitability
Rashes (photo available)	Patient encountering	The patient rarely knows if the matter is suitable for a remote appointment
Simple issues		Time pressure affects suitability
Singular contacts		Unsuitable issues can often be taken forward at remote appointments
Skin changes (photo available)

“But in the first place, it is always the attending physician who decided whether this is something that can be treated or not.”

Continuity of care overall was considered important, and the PCPs found online chat to be mostly suitable for singular contacts. It is also possible to maintain continuity of care by working remotely if the PCP works in the same region. Continuity of care is a challenge regionally and in F2F appointments and is always a challenge working part-time.

“When I only do remote work in one municipality, I would say that the continuity of care succeeds reasonably well. I have noticed that the same people come there, and many may even apply to my remote appointment. I have noticed that some people like remote services and that I meet the same names over and over again. This is probably closer to the normal work of a doctor that I have done previously.”

The PCPs felt that the patient selection succeeded mostly well, but a lack of resources elsewhere drives unsuitable patients to remote consultations. The same problems are found with regular F2F consultations. The PCPs considered whether artificial intelligence (AI) or a chatbot could direct the patients more appropriately:

“I don’t see any difference between F2F and remote patient drift. It’s just the same problem every time, F2F and remote. Patients are always directed to the wrong places where I have been working or to the wrong appointments, even in the public sector. The patients who belong in the emergency department come to a normal appointment and vice versa. It’s an eternal problem, the patient referral, no matter what the situation is. Some kind of bot should be invented to direct patients to the right place. That would be a job for some engineers.”

### Technical liabilities

The PCPs felt that the technology of remote consultations mostly functions, but when it does not, it poses a great challenge. Technical challenges affect the scheduling of remote consultations and work management and make remote consultations vulnerable.

“Just that information technology, that’s the first thing that comes to mind in this remote work. Its inoperability. If I receive a message from a colleague in the morning asking if I can start earlier because his/her application doesn’t work. It feels miserable. It interferes with his/her whole day before someone can fix it. It happens every day. Otherwise, remote work, when it goes smoothly and the tools are in order, is nice and smooth. But the tools do fail many times.”

Patients’ technical skills have an impact on the success of remote consultations, and the elderly need guidance in using remote applications.

“And then the big issue is the patient’s technical skills. It has been the biggest problem in those video consultations that the patient doesn’t know how to use them technically. They cannot turn on the sound or turn on the video, so the consultation has to be performed as a phone call.”

## Discussion

### Main findings

In this qualitative study, we found two main themes describing how PCPs perceived remote consultations: Remote consultations have an impact on the way physicians work, and remote consultations have an impact on the service system and patients.

The subthemes of the first main theme included the physicians’ new way of working, impacts on physicians’ wellbeing, and impacts on communication and physicians’ competences. The subthemes for the second main theme were the importance of smoothness of services for the patients, patient suitability, and technical liabilities.

### Findings in relation to other studies

#### The new way of working and its impact on wellbeing at work

We found that PCPs felt remote consultations brought them a new way of working when they were able to work remotely from home. Most of the participants felt that working remotely is less stressful and brings more freedom and balance to working days. The findings are similar to those found in the previous literature. Flexibility of work has been found to be positive for the physicians’ work and wellbeing [5,6, 22, 24, 30]. In two previous studies, physicians have perceived the workload as significantly lower when performing remote consultations [6, 24].

Although most of the physicians in our study perceived the workload as lower in remote consultations compared to F2F, online chat was considered to have a risk for increased workload with an unpredictable number of patients. One of the physicians noted online chat as a challenge of self-management. Similar findings have been made earlier in online chats where the pace and number of patients are unpredictable [24]. There are also other findings that digital-first approaches could increase the workload of physicians unless the initial assessment is short and a high proportion of contacts are managed without needing subsequent F2F reviews [7, 13]. Challenges with workload have also occurred when there has been less support in implementation and poor training for the use of digital tools [30].

As a downside of remote work, the participants felt that working solely remotely makes the clinical approach narrow. Combining F2F and remote work has been considered important in a prior paper [6]. Furthermore, the participants in our study missed the working community while working remotely. However, they found new ways to improve the sense of community, e.g. by using digital applications for sharing thoughts with colleagues. In a previous study, it has been shown that physicians may feel socially isolated working remotely, but depending on the job community, the opportunity to interact with colleagues can also be facilitated digitally [6].

#### Impacts on physician’s skills and communication

We found that the PCPs perceived the need for specific skills to be able to perform remote consultations adequately. Most importantly, the participants considered strong previous clinical experience in F2F consultations necessary to enable decision-making remotely, as the decisions are made with incomplete information without a physical examination. The importance of sufficient clinical experience is also in line with previous literature [5, 22, 24]. In addition, in previous studies, professionals have called for training for performing remote consultations [3, 29,30], but our participants did not bring this up in their discussions.

In addition to clinical competence, the participants perceived the need for specific communication skills regarding remote work. In the previous literature, physicians have also called for skills in communicating remotely [31–33]. The PCPs in our study noted that the patients’ capability to express themselves by writing varied and that communication by writing challenged the interpretation and understanding of terms and the transmission of empathy. They highlighted that communication in online chat requires accuracy of expression and neutral language. The risk of the bilateral misinterpretation of communication between the physician and the patient in online consultations has been noted in earlier studies [12, 24]. The loss of non-verbal clues has been shown to have an impact on communication and decision-making [4,5, 13, 24]. Forming trust and conveying empathy has been shown to require communication skills [4].

In our study, the PCPs perceived video consultation had added value to communication compared to online chat or telephone, but not to physical examination. With video, they felt it was possible to observe more nonverbal communication clues. Some physicians even felt that on video the interaction was as good as in F2F consultations, but others did not agree, e.g. with pain, psychiatric, or somatizing patients. In the previous literature, video consultations have been perceived to improve communication compared to telephone consultations and have been considered as good as F2F consultations, especially for mental health issues [13, 19]. In the previous literature, clinical examination through video has been considered challenging [13, 19].

#### Previous findings on the impact on the service system and patients

In this study, the physicians were satisfied when they were able to help patients quickly with their problems, especially in online chat. In previous studies, physicians have reported feelings of satisfaction in helping patients with their problems smoothly with telemedicine visits [5,6]. From the PCPs’ point of view, patients have been mostly satisfied with the remote consultations’ smoothness and easiness [3,4, 6, 8, 13, 15, 21, 24, 34]. In this study, the PCPs also felt that it is easier for some patients to discuss difficult issues online than F2F. Similar findings were reported by two previous studies [13, 24].

In our study, the PCPs perceived that easy access lowers the threshold for the patient to contact health care in a situation where they normally would not be able to book a F2F appointment. This phenomenon could lower the threshold of care and improve undertreatment regionally, as suggested in a previous study [24]. On the other hand, the PCPs in our study had faced a lot of non-medical advisory demands from patients in chat consultations and noted a risk of over-examining by requesting, e.g. laboratory tests. The risk of “unnecessary” health care has also been noted with digital consultations in previous studies [6, 23]. A need for careful consideration of patient selection has also previously been noted in a Swedish study, in which physicians felt that a large proportion of patients did not need to see a physician in digital consultations if limited healthcare resources were to be used most effectively [6].

Considering patient selection, we found that PCPs perceived that suitability depends on the remote consultation type and how much time they allocated to solving the issue. The PCPs perceived chat as an ideal tool for straightforward and simple cases that do not require a physical examination, as suggested previously considering remote consultations [5, 6, 13, 15, 19, 24, 30]. Furthermore, PCPs perceived that chat is suitable for the selected cases, especially mild infections, and rashes with an available photo. Previous studies have focused mainly on telephone and video consultations, and PCPs have felt skin conditions difficult to assess remotely [13]. A good-quality photo combined with the patient’s symptoms and history enable the physician to treat more issues remotely. In our study, the PCPs felt that it is possible to provide continuity of care through performing video consultations. In a previous study, video consultations were considered to facilitate patient care and safety if video consultations were provided through the same organization as the patient’s regular health centre [5]. The physicians felt that complex issues demanding clinical investigation were not suitable for remote consultations unless the patient was known, and this has been found also previously [4, 23]. Previous literature has also shown a risk for misdiagnosis with remote consultations if continuity of care is lacking and the patient history is unknown [6]. In our study, the PCPs clearly stated, that the physician always decides the suitability of the issue. To our knowledge, this has not been stated as clearly previously. Overall, case suitability to remote consultations seems to depend on the form of the consultation, time pressure, patient’s condition, and physician’s own clinical experience.

#### Technical liabilities

We found that technical liabilities are common and considered a significant barrier, and this is also noted in previous studies [6, 19]. Technical liabilities have an impact on patient safety but also on the work wellbeing of the professionals and their consultation schedules. Appropriate implementation, including the training of healthcare professionals and management on technical issues, has shown to be essential in ensuring effective and valuable clinical interventions [15].

### Strengths and limitations

One strength of this study was that the participants had broad experience with remote and F2F consultations. We had participants with a background in online chat and video consultations in all the interview groups. The conversation through the interviews was rich and liberated. The data were saturated while reading the transcripts, as the same phenomenon occurred in different interviews.

We performed semi-structured interviews that can be evaluated on the four-dimension criteria (credibility, dependability, confirmability, and transferability) to assess and ensure the validity of the study, adapted from Lincoln and Guba’s trustworthiness criteria for the qualitative study [35]. The sampling, data collection, and analysis process are reported in detail, which augments the credibility of the study. In addition, the researchers constantly discussed the analysis throughout the study. Confirmability was strengthened by focusing on the manifest content during the analysis. Dependability and transferability were evaluated.

One limitation could be that the physicians had a special interest in remote work, and they had voluntarily chosen to work in that field. This means that the participants could be early adopters possibly having positive attitudes towards remote consultations and remote work. Another limitation was that the main researcher (KK) held a supervisory position over some of the PCPs, which could have had an impact on the answers. However, we wanted to interview PCPs who had at least six months of experience performing video and/or chat consultations, and this was not possible in any other team in the company at that moment, nor in the public sector in the area. In addition, we wanted to have a purposive sample with PCPs having sufficient experience of remote consultations. Furthermore, we considered that interviewing PCPs from a competing company could have affected their answers.

## Implications for further studies and clinical practice

Our findings have implications for clinical work and further studies. Strong clinical experience and clinical skills were considered prerequisites for safe and high-quality remote work. It would be important to study more on what is strong enough clinical experience for remote consultations.

In addition, only selected complaints are considered suitable for remote consultations, and patient selection seems to be pivotal for the adequate use of resources. Digital services complement other health care services, and digitalization without added value should not be a target. The role of remote consultations as a complement and their importance in terms of strengthening continuity of care should be further studied.

The participants considered that treating suitable issues in remote consultations saves F2F appointments for those who need them – this is one matter that should be studied further. Moreover, do remote consultations save resources for F2F consultations, or do they add on demand?

From the physicians’ point of view, remote consultation could be one way to lighten stressful primary health care work. It is also necessary to pay attention to the PCPs’ well-being at work to facilitate coping with the continuous demands. Flexibility of work and a good work-life balance in different life situations could extend careers and improve workforce retention, which is one of the main challenges of health care worldwide [36]. The versatility of the work could keep jobs attractive and improve coping at work. Digital services should be designed in a way that supports the professionals’ work.

In future professional training, it is essential to stress the importance of communication and the expression of empathy in remote consultations. In addition, patients’ perspectives on remote consultations need to be studied more. In future studies, also other professionals’ perspectives, nurses, physical therapists, etc. need to be evaluated.

Moreover, the organization should have sufficient operating instructions for technical interruptions. Our study calls for the need for sufficient IT support staff and adequate technical instructions and support for patients as well.

## Conclusion

The findings of this study strengthen the previous evidence considering remote consultations. Performing remote consultations provides PCPs with a new way of working that could improve work-life balance. However, it is important to maintain sufficient clinical competence through versatile work, and we suggest that remote consultations should be only one part of the PCPs’ job description. In addition, digital consultations can make contacting healthcare smoother and easier for the patients, if the patient selection is performed carefully. Selecting suitable issues to be treated in remote consultations would help to keep the consultations safe and not overwhelm the system. Considering suitability, online chat seems suitable for singular contacts and simple issues, but remote consultations (i.e. video consultations) could provide a tool to maintain continuity of care with familiar patients.

## Supplementary Material

Attachement1_Questions.docx

## Data Availability

The data are available upon reasonable request.

## Abbreviations

F2F: Face to face
PCP: Primary care physician
