# Supplementary material for: Primary care physicians’ experiences of video and online chat consultations: a qualitative descriptive study
Source: Scand J Prim Health Care. 2024 Aug 18;43(1):47–58. doi: 10.1080/02813432.2024.2391406 (PMC12135696; doi:10.1080/02813432.2024.2391406)
Supplement: Attachement1_Questions.docx [file IPRI_A_2391406_SM6956.docx]

Appendix 1

Question frame for focus group interviews

1. What thoughts are evoked by participating in this interview?
2. Can you recall one memory from a remote consultation?
3. What kind of patients have you faced in remote consultations?
   1. What kinds of patients and health issues are suitable for remote consultations?
   2. What kind of patients are not suitable for remote consultations?
4. Compare remote consultations to face-to-face consultations.
   1. In diagnostics
   2. In treatment
   3. Advantages
   4. Disadvantages
5. What kind of notifications have you made in communicating with patients in remote consultations?
   1. How does it differ from F2F consultations?
   2. How has the communication succeeded in chat?
   3. How about with video?
6. How does the knowledge of the service system appear when performing remote consultations compared to F2F consultations?
   1. How can continuity of care be managed in remote consultations?
7. What kind of challenges have you faced in remote consultations, and how often?
   1. In technical issues
   2. In schedules
   3. In communication
   4. In consultations
8. What have you noticed with patient selection in remote consultations?
   1. Can patients assess themselves if their issue is suitable for remote consultation?
   2. How suitable for remote consultation are patients that are evaluated first by nurses and then guided to the remote consultations?
9. How has your opinion of remote consultation suitability changed during your working experience?
10. Have you noticed that easy access would increase the demand?
    1. Have you noticed issues that you normally do not face in f2f consultations?
    2. If so, have you noticed over-treatment?
11. How do remote consultations impact your wellbeing and managing in your work compared to F2F consultations?
12. What kind of training or experience is needed to perform remote consultations?
13. Do you have anything else you would like to bring up?
